# Correction to “Failure to Protect Against Myocardial Ischemia‐Reperfusion Injury With Sevoflurane Postcondioning in Old Rats In Vivo”

**DOI:** 10.1111/aas.70198

**Published:** 2026-01-28

H. Li, C. Zhou, D. Chen, N. Fang, Y. Yao, and L. Li, “Failure to Protect Against Myocardial Ischemia‐Reperfusion Injury With Sevoflurane Postcondioning in Old Rats In Vivo,” *Acta Anaesthesiologica Scandinavica* 57 (2013): 1024–1031, https://doi.org/10.1111/aas.12156.

The email address for author Lihuan Li was incorrect and should have read as either llhfw2008@163.com or goodman006@sina.com.

Additionally, the first author Huatong Li's affiliation was incorrect. The correct author affiliations should read as follows:

**Authors and Affiliations:**

Huatong Li^1,2^, Chenghui Zhou^2^, Dong Chen^2^, Nengxin Fang^2^, Yuntai Yao^2^, Lihuan Li^2^

^1^ Department of Anesthesiology, The Second Hospital of Tianjin Medical University, Tianjin, China

^2^ Department of Anesthesiology, Fuwai Cardiovascular Hospital, Chinese Academy of Medical Sciences, Peking Union Medical College, Beijing, China

We apologize for these errors.

